# Cardiac rupture complicating acute myocardial infarction: the clinical features from an observational study and animal experiment

**DOI:** 10.1186/s12872-020-01683-y

**Published:** 2020-09-10

**Authors:** Qun Lu, Ping Liu, Jian-Hua Huo, Yan-Ni Wang, Ai-Qun Ma, Zu-Yi Yuan, Xiao-Jun Du, Ling Bai

**Affiliations:** 1 Department of Cardiovascular Medicine, First Affiliated Hospital, School of Medicine of Xi’an Jiaotong University, No.277 Yanta West Road, Xi’an, Shaanxi 710061 P.R. China; 2 Experimental Cardiology Lab, Baker Heart and Diabetes Institute, 75 Commercial Road, Melbourne, Victoria 3004 Australia; 3 College of Basic Medical Sciences, Xi’an Jiaotong University Health Science Center, Xi’an, Shannxi Province P.R. China

**Keywords:** Cardiac rupture, Acute myocardial infarction, Sudden cardiac death, Prognosis, Risk factor, Reperfusion

## Abstract

**Background:**

Cardiac rupture (CR) is a fatal complication of ST-elevation myocardial infarction (STEMI) with its incidence markedly declined in the recent decades. However, clinical features of CR patients now and the effect of reperfusion therapy to CR remain unclear. We investigated the clinical features of CR in STEMI patients and the effect of reperfusion therapy to CR in mice.

**Methods:**

Two studies were conducted. In clinical study, data of 1456 STEMI patients admitted to the First Hospital, Xi’an Jiaotong University during 2015.12. ~ 2018.12. were analyzed. In experimental study, 83 male C57BL/6 mice were operated to induce MI. Of them, 39 mice were permanent MI (group-1), and remaining mice received reperfusion after 1 h ischemia (21 mice, group-2) or 4 h ischemia (23 mice, group-3). All operated mice were monitored up to day-10. Animals were inspected three times daily for the incidence of death and autopsy was done for all mice found died to determine the cause of death.

**Results:**

CR was diagnosed in 40 patients: free-wall rupture in 17, ventricular septal rupture in 20, and combined locations in 3 cases. CR presented in 19 patients at admission and diagnosed in another 21 patients during 1 ~ 14 days post-STEMI, giving an in-hospital incidence of 1.4%. The mortality of CR patients was high during hospitalization accounting for 39% of total in-hospital death. By multivariate logistic regression analysis, older age, peak CK-MB and peak hs-CRP were independent predictors of CR post-STEMI. In mice with non-reperfused MI, 17 animals (43.6%) died of CR that occurred during 3–6 days post-MI. In MI mice received early or delayed reperfusion, all mice survived to the end of experiment except one mouse died of acute heart failure.

**Conclusion:**

CR remains as a major cause of in-hospital death in STEMI patients. CR patients are characterized of being elderly, having larger infarct and more server inflammation. Experimentally, reperfusion post-MI prevented CR.

## Background

Cardiac rupture (CR) consists of free wall rupture (FWR) and ventricular septum rupture (VSR), and is a lethal mechanical complication of acute myocardial infarction (MI) [1], the incidence of CR was between 7 to 20% in ST-elevation MI (STEMI) patients during 1970s to 1990s [2, 3], when CR was reported to occur either early after the onset of MI (type I or II, approximately 55%) or during the sub-acute phase accompanied with overt cardiac remodeling (type III, 45%) [3]. Factors including old age [4], female gender [4], and large infarct size [2] were found to be associated with the risk of CR. With the routine use of thrombolytic drugs and revascularization therapy including percutaneous coronary intervention (PCI), the incidence of CR has decreased to the current approximately 1% [3, 5], attributable to thrombolytic [6, 7] and reperfusion therapies [1]. Primary PCI (pPCI) as the standard treatment for MI has also remarkably reduced the in-hospital mortality and improved the long-term outcomes.

Nevertheless, current literature indicates that CR remains as an emergency of modern cardiology contributing to the total in-hospital mortality in MI patients [3]. It is unclear on the clinical features of CR in the era of reperfusion therapy, a knowledge essential for the evaluation and timely intervention of CR events and preventive procedures. There has been lack of evidence for a causal relationship between reperfusion therapy and reduced risk of CR. Accordingly, the aim of this study was to investigate the incidence, associated risk factors, timing of occurrence, and clinical outcomes of this complication in acute MI patients in the PCI era and influence of reperfusion in MI mice.

## Methods

This study was done within both MI patients and animal experiments.

### Patients with acute MI

A retrospective medical record review study was conducted using the electronic patient record system of the First Affiliated Hospital of Xi’an Jiaotong University in Shaanxi province. We screened the records of patients who were admitted to the CCU of the study hospital during December 2015 and December 2018 and identified patients with ST-elevation MI (STEMI). Exclusion criteria included patients with non-STEMI, unstable angina, or recent or remote MI. This protocol was approved by the ethics committee of the First Affiliated Hospital of Xi’an Jiaotong University (Shaanxi 710,061, China) and was in accordance with the Helsinki Declaration’s guidelines. Informed consent was obtained for all participants and families.

### Animals

Male C57BL/6 mice were introduced from Jackson Laboratory (USA) and used at the age of 12–15 weeks. Mice were housed in standard conditions with food and water provided ad libitum in a 12 h/12 h light/dark cycle. All procedures used were approved by a local animal ethics committee in compliance with the Australian Code for the Care and Use of Animals for Scientific Purposes (8th edition) and the ARRIVE guidelines.

Mice were anesthetized using the mixture of ketamine/xylazine/atropine (20/100/1.2 mg/kg, respectively) and put on a heated pad. As we previously described [8], open-chest surgery was done to induce coronary artery occlusion (CAO). Animals were randomized into 3 groups subjecting to either permanent CAO (group-1), ischemia-reperfusion (IR, group − 2 and − 3). Reperfusion was done following CAO lasting 1 h (group-2) or 4 h (group-3). All operated mice were monitored up to day-10. Animals were inspected three times daily for the incidence of death and autopsy was done for all mice found died to determine the cause of death. After this study, all mice were euthanized with pentobarbital overdose and autopsied to confirm presence of MI.

To get insight into influence by reperfusion on CR risk, we measured infarct size in a separate batch of mice. Mice were operated to induce CAO and then randomly allocated into 3 groups subjected to non-reperfused MI or IR after an ischemic period of 1 h or 4 h. Infarct size was determined 48 h post-surgery using the established dual-staining method, as we previously described [9]. In brief, at the end of reperfusion, the heart was injected via aortic cannulation with 5% Evans blue, followed by sections of the left ventricle (1 mm in thickness) and staining with 1.5% triphenyltetrazolium chloride (TTC, 30 min, 37 °C). Images of LV sections were acquired digitally and the risk zone and infarct zone were determined using Image J software and infarct size was expressed as percentage of risk zone.

### Diagnosis of ST-elevation myocardial infarction

According to the AHA/ACCA [10] and ESC [11] guidelines for STEMI patients, diagnosis of STEMI was based on the concurrence of chest pain or symptoms compatible with acute heart failure or unexplained syncope, ST-segment elevation ≥1 mm in 2 inferior or lateral leads or ≥ 2 mm in ≥2 precordial leads and elevation of cardiac biomarkers (CK-MB or troponin-T).

### Diagnosis of cardiac rupture

**Fig. 1:**
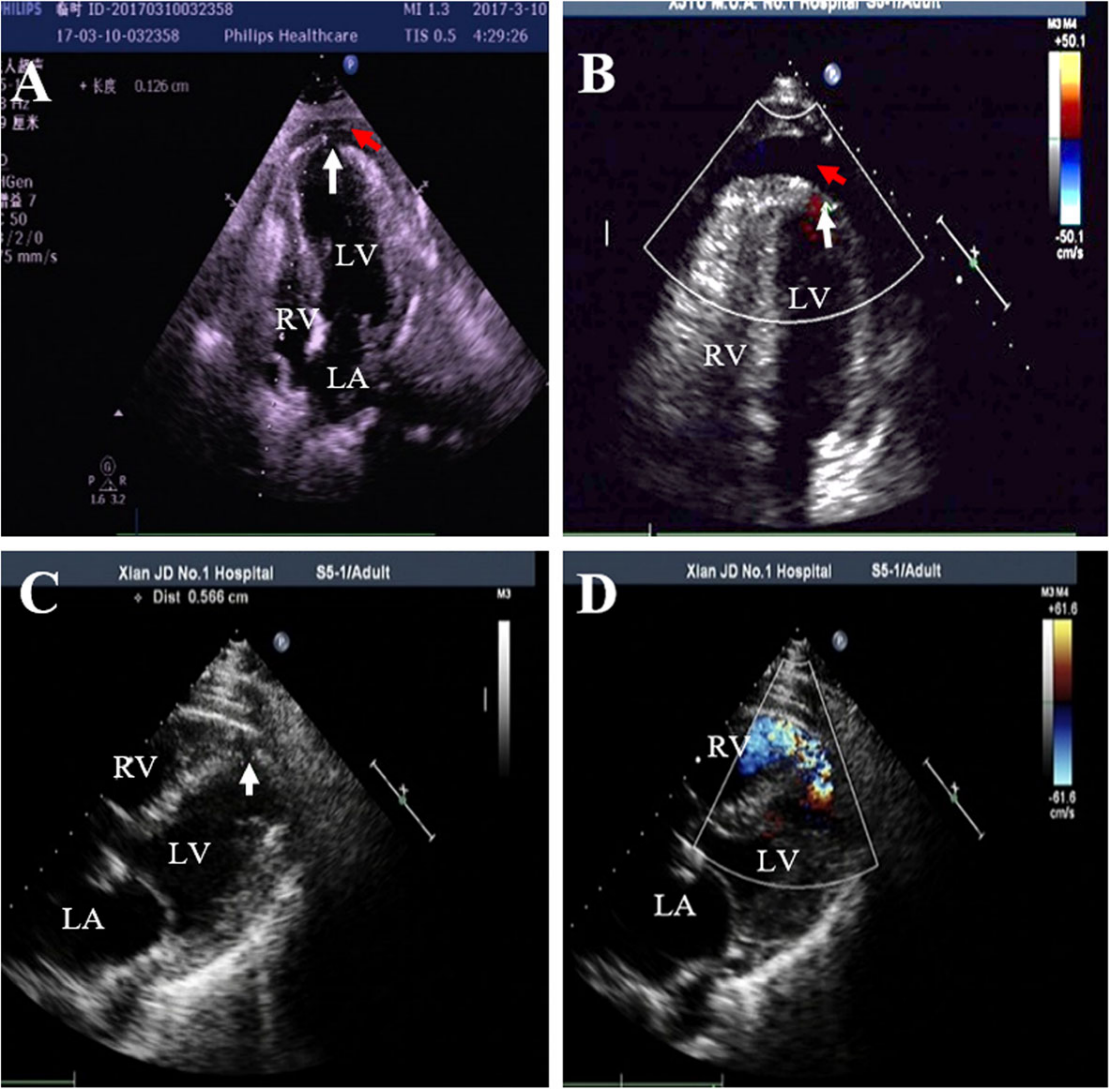
Representative transthoracic echocardiographic images from patients with cardiac rupture. **a** 2-D image revealed discontinuity of the left ventricular (LV) wall (white arrow) and pericardial effusion (red arrow). **b** Color Doppler image demonstrated shunting of blood flow from the LV to the pericardium (white arrow) and presence of pericardial effusion (red arrow). **c** ventricular septum discontinuity (white arrow) in 2-D image. **d** shunting of blood from the LV to the right ventricle (RV, red arrow) in Color Doppler image; LA = left atrium; RA = right atrium

Among STEMI patients, sing CCU-quipped Phillips iE33 system, echocardiography was performed at day-1 after admission, as well as before hospital discharge or when the following conditions were observed during the infarct evolution: hypotension, syncope, chest pain, ECG changes, severe arrhythmias or conduction disturbances. Patients were placed on supine or on left recumbent position. 2-Dimensional echocardiograms were acquired using Phillips iE33 ultrasound system with S5–1 probe. Images were obtained in the standard parasternal long- and short-axes, apical and subcostal 4-chamber views. Color-Doppler echocardiography was used in conjunction with 2-dimensional echocardiography. All images were recorded on videotape and analyzed by at least two echocardiography specialists. Diagnosis of CR was based on echocardiographic findings and clinical manifestations. FWR was diagnosed by the presence of echo-signal free space of the free wall myocardium or presence of pericardial effusion (Fig. 1a and b), Color Doppler detected blood flow shunt between the ventricle and the pericardium (Fig. 1b) when patients developed sudden onset of cardiogenic shock, conscious disturbance, and pulseless electric activity (electro-mechanic dissociation) after being in a stable condition [12]. VSR was suggested by physical examination of strong cardiac murmur and diagnosed by echocardiography as presence of echo signal-free pace of the ventricular septum (Fig. 1c and d) and Color Doppler detected blood flow signal across the ventricular septum (Fig. 1d) [12].

In animal study, CR diagnosis was confirmed by autopsy findings of presence of a large amount of blood clot around the heart and in the chest cavity, a perforation of the infarcted wall [8].

### Clinical measurements

Investigators and a trained interviewer collected all the clinical data. Potential predictive variables evaluated in this study included demographic characteristics (age and sex), classical cardiovascular risk factors (smoking, hypertension, diabetes mellitus and alcohol consumption), characteristics of MI (localization, culprit artery, numbers of diseased vessels) and Killip’s classification. Smoking was defined as smoking cigarettes within 1 month of the index admission. Alcohol consumption was defined as drinking > 30 mL ethanol per day in men and > 15 mL ethanol per day in women. Hypertension was defined as a cuff blood pressure ≥ 140/90 mmHg and/or the current use of antihypertensive medications. Diagnosis of diabetes was confirmed as plasma fasting glucose was ≥7.0 mM (or the 2-h postprandial glucose was > 11.1 mM) and /or there was current use of anti-diabetic medication. Killip classification was assigned for the presence and severity of heart failure for STEMI patients according to AHA/ACCA [10] and ESC guideline [11]. Infarct location was determined by electrocardiogram and echocardiography. Selective coronary angiograms were obtained using the Judkins or Sones technique. The coronary arteries were analyzed by routine angiogram. On the basis of luminal stenosis ≥50% as significant coronary stenosis, the number of coronary arteries with lesion was determined. Information also included socio-economic features of patients as resident of urban or countryside. Surrounding country-level regions, including counties and county-level cities, were then designated as rural area.

### Laboratory parameters

For measurements of hemoglobin (HB), blood cell counts or high-sensitivity C-reactive protein (hs-CRP), blood was collected via the median cubical vein using EDTA-containing tubes at admission and daily afterwards. HB, white blood cell (WBC) and neutrophils were measured with automated cell counters via standard techniques by HST201 (Sysmex, Japan). hs-CRP was measured by high-sensitivity particle-enhanced immunoturbidimetric method using BN II (Siemens, Germany). Blood was also drawn into sodium citrate-containing tubes, at admission and then daily after a 12-h overnight fasting for measurements of fibrinogen, D-dimer and fibrinogen degradation products (FDP), by using the latex agglutination test (Sysmex CA − 7000, Sysmex, Japan). To detect the peak of creatine kinase-MB (CK-MB), blood was collected into tubes containing no anticoagulant at admission and then every 6 h after symptom onset for 24 h, CK-MB were determined by the spectrophotometric method using the Olympus AU640 Clinical Chemistry analyzer (Olympus Diagnostica, Hamburg, Germany).

### Treatment and evaluation of patient outcomes

All patients were administered loading doses of aspirin 300 mg and clopidogrel 600 mg or ticagrelor 180 mg, followed by maintenance doses of aspirin 100 mg, clopidogrel 75 mg or ticagrelor 180 mg daily. All patients received β-blockers, angiotensin converting enzyme inhibitors (ACEI) or angiotensin receptor blockers (ARB) and statins according to the AHA/ACCA [10] and ESC STEMI guidelines [11], unless there were contraindications to these drugs. All patients were administered pPCI or delay PCI based on clinical manifestation according AHA/ACCA [10] and ESC STEMI guidelines [11]. All CR patients were suggested equally to reperfusion, intra-aortic balloon pumping (IABP), extracorporeal membrane oxygenation (ECMO), percutaneous closure or surgical repair, if patients had not contraindications. The primary outcome was mortality, defined as deaths of any cause in-hospital or within 60 days. In-hospital mortality was obtained from hospital medical records. Patients were also followed up for information of 60-day mortality by interview or telephone communication with these patients or their families.

### Statistical analyses

Analyses were performed using SPSS version 13.0. Normally distributed values are presented as mean ± SD, and differences between groups were determined using ANOVA followed by Student’s t-test. Variables with a skewed normal distribution are presented as medians (interquartile range), and between-group differences for these variables were determined using Rank-Sum test. Categorical variables are presented as percentages, and the differences between groups were tested using Chi-square test. The logistic model was used to evaluate the associations between cardiac rupture and variables. Odds ratios (OR) and 95% confidence intervals (CI) were calculated. Survival estimates were generated using Kaplan-Meier method. Significance was defined at the 5% level using a two-tailed test.

## Results

### Incidence and time-course of cardiac rupture

**Fig. 2:**
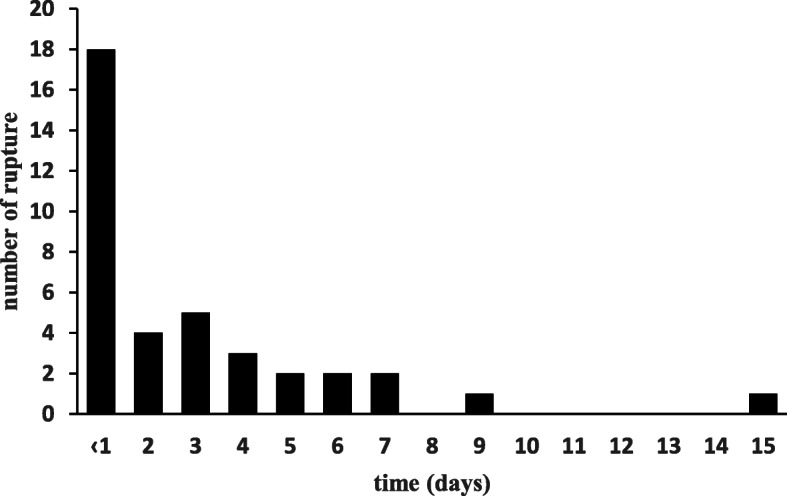
The times from symptom onset to cardiac rupture diagnosis

During December 2015 to December 2018, a total of 5844 consecutive patients were admitted to the CCU. Of them, 2568 patients were excluded according to the exclusion criteria and the remaining 1456 patients with confirmed STEMI diagnosis were included in this retrospective study. There were 40 patients diagnosed with CR (Table 1), VSR occurred in 20 cases, FWR in 17 cases and combined location in 3 cases. 19 patients had CR before arriving the hospital with estimated symptom-to-CR interval of 4 h to 15 days. CR occurred within hospital in 21 patients giving the in-hospital incidence of CR 1.4% (21/1456). In 9 patients CR developed post-pPCI, and 2 cases received thrombolytic therapy. As shown in Fig. 2, 18 patients (45%) developed CR within 24 h after chest pain symptoms onset. And 16 patients (40%) occurred CR during 2–6 days after symptoms onset, and another CR occurred in another 4 (10%) patients during 7–15 days after STEMI.

**Table 1:** Clinical characteristics of cardiac rupture

Case	gender	Age (y)	Location of AMI	Rupture Site	Diagnosis method	TT	PCI	surgical repair	percutaneous closure	IABP/ECMO	Outcome (death)	Onset MI to Death (Days)
1	M	64	A	FWR	UCG	Yes	Yes	No	No	No	No	
2	M	74	A	FWR	UCG	No	Yes	No	No	No	No	
3	F	79	A	VSR	UCG	No	Yes	No	No	Yes	Yes	31
4	M	83	I	VSR	UCG	No	No	No	Yes	Yes	No	
5	M	83	I	FWR + VSR	UCG	No	Yes	No	No	Yes	Yes	6
6	M	69	A	VSR	UCG	No	No	Yes	No	Yes	No	
7	M	68	A	VSR	UCG	No	Yes	No	No	No	Yes	4
8	F	65	A	FWR	UCG	No	No	No	No	No	Yes	1.0
9	F	87	A	FWR	UCG	No	No	No	No	No	Yes	0.4
10	F	67	A	VSR	UCG	No	Yes	No	No	No	Yes	1.2
11	M	69	I	FWR	UCG	No	No	No	No	No	Yes	0.4
12	F	76	I	VSR	UCG	No	Yes	No	No	No	Yes	7
13	F	58	A	FWR + VSR	UCG	No	No	No	No	No	Yes	0.3
14	M	78	A	FWR	UCG	No	No	No	No	No	Yes	2.3
15	M	75	A	VSR	UCG	No	Yes	No	No	Yes	Yes	13
16	M	46	A	VSR	UCG	No	Yes	No	No	No	No	
17	F	72	A	FWR	UCG	No	No	No	No	No	Yes	0.2
18	M	59	A	VSR	UCG	No	Yes	Yes	No	Yes	Yes	53
19	M	73	A	VSR	UCG	No	No	No	Yes	Yes	No	
20	M	76	A	VSR	UCG	No	No	No	No	Yes	Yes	3.4
21	F	76	A	VSR	UCG	No	Yes	No	No	No	Yes	10
22	F	68	I	FWR	UCG	No	Yes	No	No	No	Yes	3.4
23	M	63	I	FWR	UCG	No	Yes	No	No	No	Yes	6.2
24	F	63	A	VSR	UCG	No	Yes	No	No	Yes	Yes	15.5
25	F	66	A	VSR	UCG	No	Yes	Yes	No	Yes	No	
26	F	68	A + I	FWR + VSR	UCG	Yes	No	No	No	Yes	Yes	10.7
27	M	59	A	FWR	UCG	No	No	No	No	Yes	No	
28	F	83	A	FWR	UCG	No	No	No	No	No	Yes	0.4
29	M	49	I	VSR	UCG	No	Yes	Yes	No	Yes	No	
30	M	55	A + I	FWR	UCG	No	No	No	No	No	No	
31	M	78	A	FWR	UCG	No	No	No	Yes	No	Yes	3.2
32	F	66	A	FWR	UCG	No	No	No	Yes	No	Yes	3
33	M	61	A	FWR	UCG	No	No	No	No	No	Yes	4.4
34	M	82	I	FWR	UCG	No	No	No	No	Yes	Yes	3
35	M	72	A	VSR	UCG	No	Yes	No	Yes	No	No	
36	M	53	A	FWR	UCG	No	No	No	Yes	No	Yes	0.4
37	F	60	A	VSR	UCG	No	Yes	No	Yes	No	No	
38	F	51	A	VSR	UCG	No	Yes	No	Yes	No	No	
39	F	73	^a^	VSR	UCG	No	No	Yes	No	No	No	
40	M	58	^a^	VSR	UCG	No	No	Yes	No	No	No	

*A* anterior, *AMI* acute myocardial infarct, *ECMO* extracorporeal membrane oxygenation, F female, L lateral, FWR free wall rupture, *IABP* intra-aortic balloon pumping, *I* inferior, *M* male, *PCI* percutaneous coronary intervention, *TT* thrombolytic therapy, *UCG* Ultrasonic Cardiogram, *VSR* ventricular septum rupture

^a^ no information available

### Characteristics of cardiac rupture

Relevant patient baseline characteristics are summarized in Table 2. 45% of CR patients admitted to hospital within 12 h whilst 12.5% of CR patients arrived to hospital during 12–24 h after the onset of symptom, and the ratio of patients who arrived to hospital within 12 h after symptom was higher in CR patients than CR-free patients (Table 2).

**Table 2:** Baseline clinical characteristics in 925 STEMI patients with and without CR

	Non-rupture (*n* = 885)	Rupture (*n* = 40)	*P* value
Age (year)	58.5 ± 11.7	68.2 ± 10.1	< 0.001
Male gender (%)	731 (82.6%)	23 (57.5%)	0.002
Socio-economic feature (rural area)	446 (50.4%)	17 (42.5%)	0.041
Symptom-to-admission interval			0.017
< 12 h	324 (36.6%)	18 (45.0%)	
12 ~ 24 h	41 (4.6%)	5 (12.5%)	
> 24 h	520 (58.8%)	8 (42.5%)	
Previous history			
Diabetes	127 (14.4%)	12 (30.0%)	0.012
Hypertension	380 (42.9%)	26 (65.0%)	0.008
Alcohol consumption	266 (30.1%)	0 (0%)	< 0.001
smoking	587 (66.3)	12 (30.0)	< 0.001
Myocardial infarction	53 (6.0%)	0 (0%)	0.162
Angina pectoris	237 (27.0%)	6 (15.0%)	0.101
Coronary surgery	24 (2.7%)	1 (2.5%)	0.936
Physical examination at admission			
Systolic blood pressure (mmHg)	123 ± 20	110 ± 39	0.001
Diastolic blood pressure (mmHg)	77 ± 13	72 ± 25	0.047
Heart rate (bpm)	76 ± 14	83 ± 31	0.004
Killip class			0.002
I	552 (62.4%)	11 (45.8%)	
II	260 (29.4%)	6 (15.0%)	
III	43 (4.9%)	3 (7.5%)	
IV	30 (3.4%)	4 (10.0%)	
MI localization			
anterior	359 (40.6%)	26 (65%)	0.002
inferior	389 (44%)	8 (20%)	0.003
Infarct-related coronary artery			0.417
Left anterior descending artery	429 (56.6%)	17 (68%)	
Left circumflex artery	58 (7.7%)	2 (8%)	
Right coronary artery	266 (35.1%)	6 (24%)	
Left main trunk	5 (0.7%)	0 (0.0%)	
Number of stenosed vessel			0.854
1	202 (26.6%)	4 (21.1%)	
2	269 (35.5%)	7 (36.8%)	
3	287 (37.9%)	8 (42.1%)	

Data are mean (SD) or n (%) unless otherwise stated. Killip class for CR patients was obtained prior to onset of rupture (*n* = 24 in rupture group). Among all STEMI patients, 773 patients underwent coronary angiography

Patients with CR were older, more likely to be female and had history of hypertension and/or diabetes mellitus and lower blood pressure (Table 2). CR groups also had lower incidence of previous MI and previous PCI (Table 2). The incidence of angina prior to the current MI was comparable between CR and CR-free groups.

### Clinical biochemical or angiographic findings

**Fig. 3:**
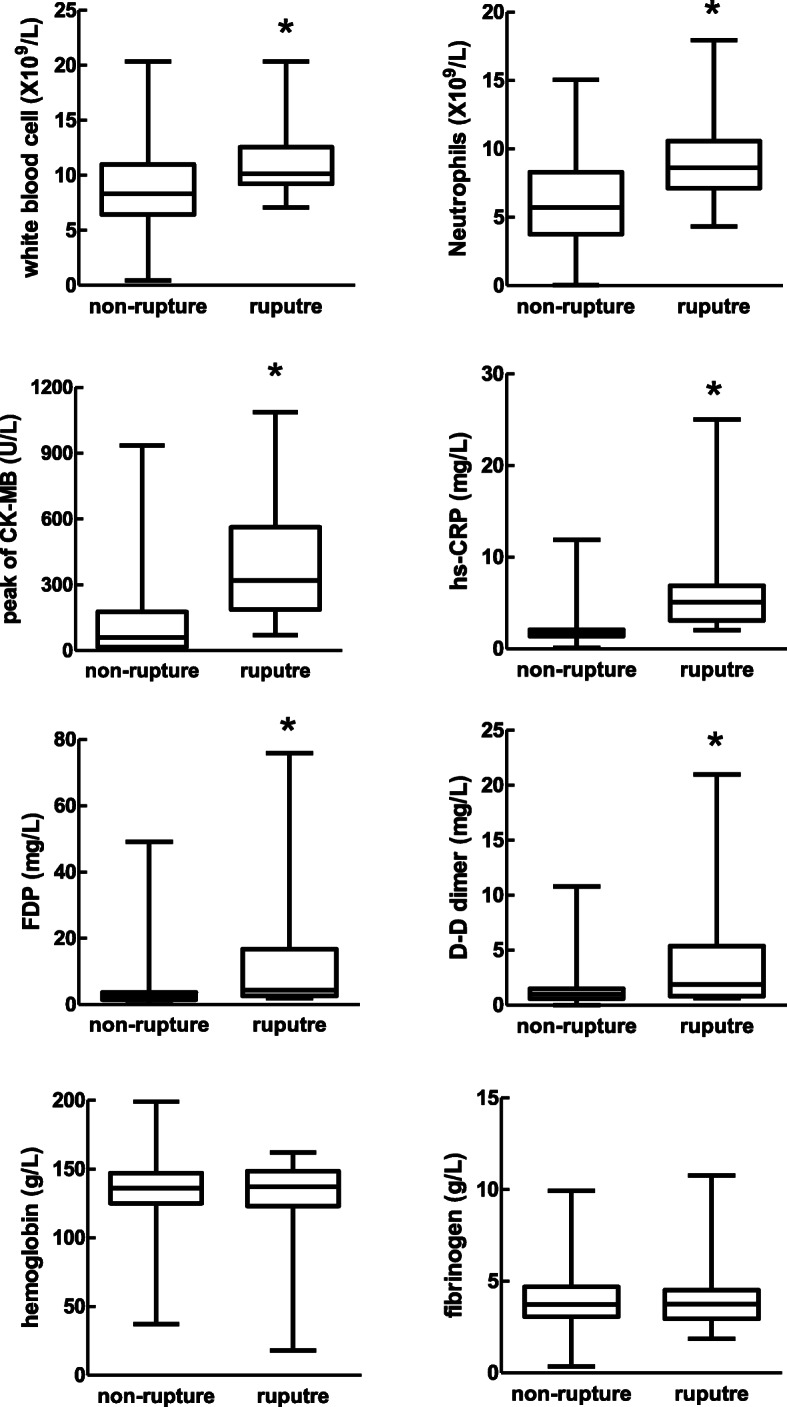
Comparison of laboratory parameter between STEMI with or without cardiac rupture. CK-MB: creatine kinase-MB, hs-CRP: high sensitive C-reactive protein, FDP: fibrinogen degradation product, WBC: white blood cell. *P < 0.05 vs. STEMI without CR. Note: *n* = 11 in rupture group for peak CK-MB and peak hs-CRP

Patients with CR exhibited higher counts of WBC and neutrophils at admission, and higher peak levels of CK-MB and hs-CRP, and higher levels of D-D dimer and FDP **(**Fig. 3**)**. There was a significant and positive correlation between peak CK-MB and peak hs-CRP (r = 0.1991, *P* < 0.001, *n* = 896). Of 40 CR patients, 19 cases (47.5%) underwent coronary angiography. There was no significant difference in patients without or with CR in characteristics of coronary lesion (Table 1).

### Risk factors associated with CR

**Fig. 4:**
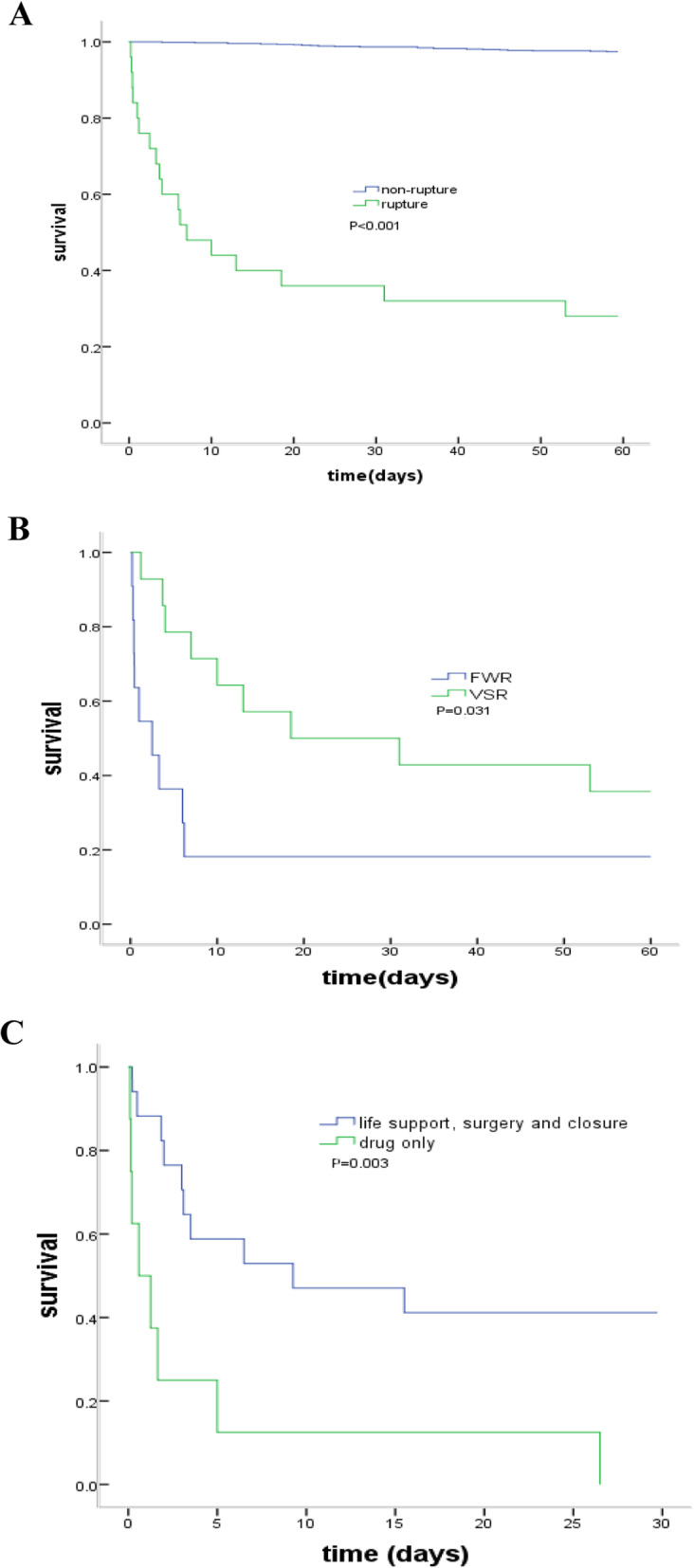
Multivariate logistic regression analysis of risk factors and the incidence of cardiac rupture. CK-MB: creatine kinase-MB; hs-CRP: high sensitive C-reactive protein

By multi-variables logistic regression models, age, female gender, peak CK-MB, peak hs-CRP and rural area were independent factors entered the regression model with OR between 2.49 to 12 (Fig. 4).

### Therapy and prognosis of patients with CR

Of 885 CR-free STEMI patients, 293 patients underwent pPCI (32.2%) and 480 patient received delay PCI (54.2%). Among patients with CR, 12 cases underwent pPCI and 7 patients underwent delay PCI, with the rest 21 patients (52.5%) did not receive PCI, a percentage higher than other STEMI patients without CR (*n* = 112, 12.7%). Regarding the medications used, the proportions of the use of aspirin (*P* = 0.027), P2Y12 inhibitor (*P* < 0.001), statin (*P* < 0.001), β-blocker and ACEI/ARB (*P* = 0.027) were significantly lower in the patients with CR (Table 3). Except for medications and PCI, CR-related treatment was also applied to our CR patients including IABP (*n* = 14), ECMO (*n* = 2), open-chest surgery for VSR (*n* = 6), or device closure therapy for VSR (*n* = 8).

**Table 3:** Treatment characteristic in STEMI patients with or without cardiac rupture

	Non-rupture (*n* = 885)	Rupture (*n* = 40)	*P* value
**Drugs**			
Aspirin	858 (97.0%)	33 (82.5%)	0.006
P2Y12 inhibitor	850 (96.0%)	29 (72.5%)	< 0.001
ACEI/ARB	793 (89.6%)	13 (32.5%)	< 0.001
β-blocker	794 (89.7%)	15 (37.5%)	< 0.001
Statins	823 (93.0%)	13 (32.5%)	< 0.001
**PCI type**			< 0.001
primary PCI	283 (32.0%)	12 (30%)	
Delay PCI	475 (53.7%)	7 (17.5%)	
Non-PCI	127 (14.3%)	21 (52.5%)	

Data are n (%). *ACEI/ARB* angiotensin converting enzyme inhibitors or angiotensin-II receptor blockers, *PCI* percutaneous coronary intervention

**Fig. 5:**
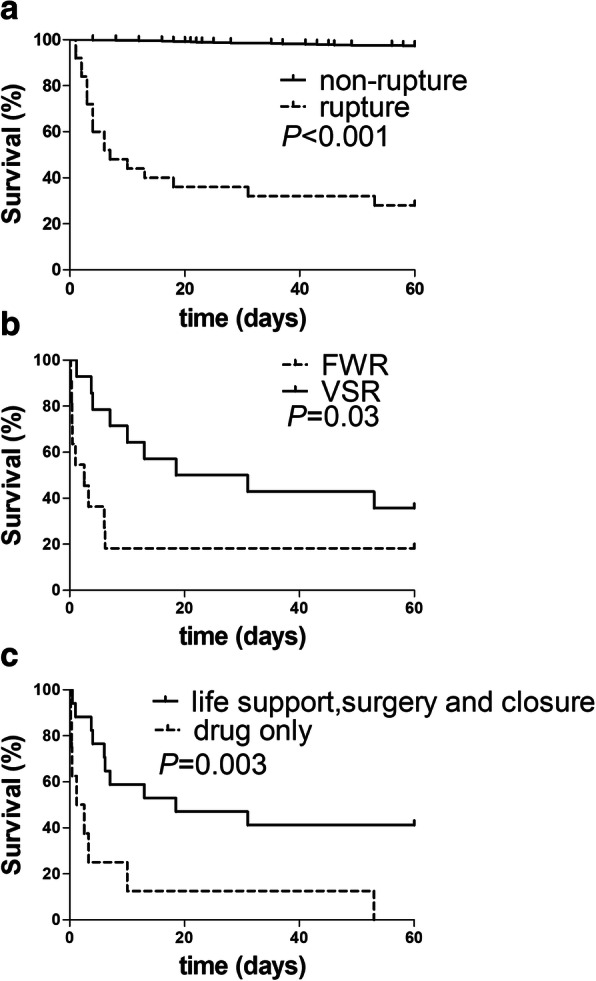
Survival curves of patients with cardiac rupture. **a** compared between in STEMI patients with and without CR. **b** compared between FWR group and VSR group. **c** compared between drug treatment only and life support, surgery and closure in CR. FWR: free wall rupture, VSR: ventricular septum rupture

Among 910 patients, a total of 48 patients died in-hospital and 61 patients died within 60 days. Of 40 CR patients, 2 patients (57.5%) died in hospital and 25 (62.5%) died within 60 days, contributing to 47.9% (23/48) in-hospital deaths and 41.0% (25/61) 60-day deaths. Other causes of in-hospital death were heart failure (10, 20.8%), arrhythmias (9, 18.8%) or cardiogenic shock (6, 12.5%). Similarly, non-CR reasons for 60-day deaths consisted of heart failure (17, 27.9%), arrhythmias (12, 19.7%) or cardiogenic shock (7, 11.5%). The post-STEMI survival was much lower in CR patients compared with CR-free patients (Fig. 5a). By survival analysis, open-chest surgery, hemodynamics support or device closure therapy may be associated with improved prognosis (Fig. 5b). The death rate was higher in patients with FWR was than these with VSR (Fig. 5c).

### CR time-course and influence of reperfusion in mice

A total of 83 mice were operated with 39 mice were allocated in group-1, 21 mice in group-2 and 23 mice in group-3. There were 7 mice died within 24 h after surgery due to surgical related reasons (bleeding, poor recovery from the procedure, *n* = 5) or acute heart failure (*n* = 2). The remaining mice (*n* = 36 for group-1, *n* = 19 for group-2, and *n* = 21 for group-3) were included in observation of CR up to day-10 after surgery. At the end, all mice were euthanized and presence of cardiac infarct was confirmed by autopsy.

**Fig. 6:**
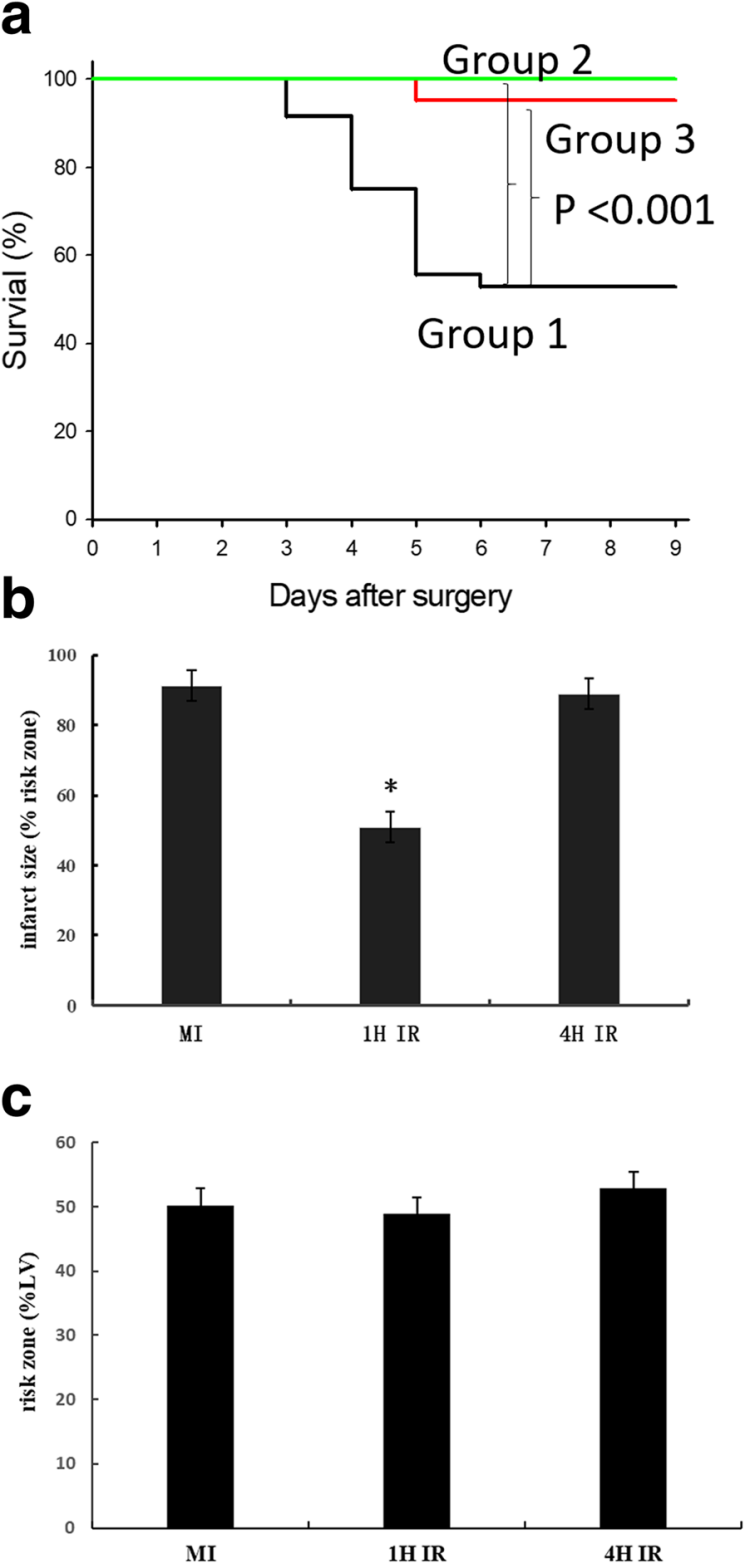
Effect of reperfusion on the incidence of CR and infarct size in mice. **a** Comparison of survival in mice with permanent coronary artery occlusion (Group-1, *n* = 36), or reperfusion following 1-h (Group-2, *n* = 19) or 4-h (Group 3, *n* = 21) ischemia. **b** influence of reperfusion on infarct size in relation to ischemic duration. Note that infarct size was significantly reduced by early (i.e. 1-h ischemia) but not delayed (4-h ischemia) reperfusion. Infarct size date are mean ± SEM. The group size was 6–8. *P < 0.01 vs. non-reperfused MI or 4-h IR group

There were 17 deaths in group-1 occurring during day 3–6 post MI (Fig. 6a). Autopsy indicated all deaths due to LV free wall rupture with large amount of blood clot in the chest cage, albeit co-existence of acute heart failure was identified in 3 mice (i.e. pulmonary congestion, chest pleural effusion). In the two IR groups, one mouse died of acute heart failure at day 5 post surgery (Group-3) and the rest of mice survived to the end of experiment without onset of CR (*P* < 0.001 for either of group-2/3 vs group-1).

Influence of reperfusion on infarct size was determined in a separate cohort of mice. At 48 h after non-reperfused MI or IR (with 1- or 4-h ischemia, *n* = 6–8 per group), the risk zone was comparable among groups (Fig. 6b). In non-reperfused MI group, almost entire ischemic myocardium become necrotic, whereas infarct size was significantly smaller in mice with 1-h IR. However, infarct size in 4-h IR mice was comparable to that of MI group (Fig. 6b).

## Discussion

This retrospective study leads to the following main findings. First, the current in-hospital CR incidence is 1.4% in STEMI patients, but the real incidence is likely to be higher considering the pre-hospitalization onset of CR since a high proportion of CR occurred within day-1 after STEMI. Second, the in-hospital and 60-day mortality of CR patients remains very high. Third, open-chest surgery, life support or device closure therapy are superior to medications to improve the prognosis of CR patients. Forth, the risk of CR is higher if STEMI patients are elderly, with large infarct size and high levels of inflammatory parameters. Finally, reperfusion completely eliminated CR in mice with MI.

The overall incidence of CR reported in the literature prior to the pPCI era was 10–20% [2], and the incidence of CR reported over the recent 10 years is around 1% [1, 13, 14]. In the current study, the in-hospital incidence of CR was 1.4% in STEMI patients. Practically, the true incidence of CR is difficult to reach, as indicated by our study revealing that 19/40 patients developed CR at admission. Such a high proportion of patients with early onset of CR highlights that CR remains a major challenge to modern cardiology and forms one of the main reasons of sudden cardiac death post-MI. A further hurdle added to this challenge is the current very low autopsy rate. In a recent study, Chen et al analyzed 11,234 STEMI patients from the 7 major hospitals in China and found that the incidence of CR was between 1 and 4% among these hospitals [15]. Early reperfusion therapy could be the major factor responsible for the decline in the incidence of CR [1]. The results of mouse study provide a strong experimental evidence for the overt reduction of rupture in the last few decades with the initially increasing and currently routine use of primary PCI^1^.Although ACC/AHA [10] and ESC [11] Guidelines recommend reperfusion therapy within the first 12 h after symptom onset in all STEMI patients, some STEMI patients could not receive reperfusion therapy due to various reasons including delayed admission. Unfortunately, study has revealed that the proportion of patients in China who did not receive pPCI has not significantly improved over the last decade [15]. Low proportion of reperfusion therapy is likely one of the reasons for the onset of CR in-hospitalization.

Clinical reports prior to the reperfusion therapy era described the frequency of CR as two peaks: within 24 h and during 6–14 days after STEMI with nearly even proportion [2, 16]. In our population study, whilst the early peak of CR remains, the late peak of CR seems blunted. Based on the Becker classification of CR [17], early CR is mainly type-I or type-II, whereas late rupture were type-III associated with significant wall thinning and ventricular remodeling. The clinicopathological features of early and late rupture are different [18]. Early phase rupture is characterized by an abrupt slit-like tear in the infarcted myocardium, while late phase rupture exhibits infarct expansion and wall thinning [18].

While some studies indicated that reperfusion therapy is only associated with reduced incidence of late CR while showing limited benefit on the early CR [16, 19], it is generally agreed that early reperfusion is able to reduce the rupture incidence [20]. In our CR patient study, 9 patients had early CR after pPCI. In contrast, our mouse study revealed that early or delayed reperfusion similarly abolished CR, albeit infarct size was reduced by early but not by delayed reperfusion. The benefit of delayed reperfusion is likely attributable to protection of other myocardial cellular populations and/or extracellular matrix [21]. Thus, the influence of reperfusion therapy in patients with MI requires further investigation. Whilst the mechanism for the strong protection against CR, seen in mice with MI, remains to be illustrated. The infarct size might be reduced by reperfusion after 1 h ischemia in mice study [8], but not if ischemia last for 4 h, it is clear that the protection associated with reperfusion not only pertain to cardiomyocytes, but also to non-cardiomyocyte matrix tissues within the ischemic zone, which provides tensile strength of the infarct wall [8, 22].

Our study showed that the mortality of CR remained to be very high (64% within hospital and 72% within 60 days) relative to the earlier reports showing 100% mortality for FWR [2] and approximately 90% for VSR without treatment [23], albeit the mortality of patients underwent surgery for correction of VSR varied between 20 and 60% [12, 24]. Management of CR patients is complex and might require a variety of therapeutic approaches, including pharmacologic (include ACEI, β-blocker, intravenous nitrates, and hydralazine) and device-based therapies to achieve afterload reduction and hemodynamic stabilization. Whilst medical therapy and non-pharmacologic methods may only stabilize CR patients, the treatment of choice is closure of rupture site by surgical and catheter-based means [25, 26]. The outcome of operated CR patients is closely related to their hemodynamic state prior to the surgery. Emergency surgery for CR has been limited in our hospital due to the fact that patients with CR are often in extremis prior the surgery and many died suddenly. On the other hand, currently the percutaneous devices to primarily close VSR are only applicable to selected cases with simple defects (e.g. VSR less than 15 mm in diameter) with the optimal time approximately 3 weeks following MI. In our cohort, only 8 out of 40 CR patients were suitable for percutaneous closure at 3 weeks after rupture occurred and they both survived over 60 days. Unfortunately, we experienced a high percentage of CR patients who refused further treatment because of reasons including critical conditions or high expenses.

In the current study, we revealed several independent risk factors of CR including female gender, old age, lower MI or angina history or higher heart rate, findings similar to earlier reports [2, 4, 27, 28]. Our study also added mechanistic insight by showing that both infarct size and the extent of inflammation are underlying factors for the onset of CR. The enzymatic index of infarct size, peak CK-MB [29], was one of the independent risk factors of CR in patients with STEMI. However, caution is required since many patients died before they arrived hospital with their infarct size was difficult to assess due to lack of serial measure of CM-MB and hence such relationship between infarct size and the risk of early onset of CR remains elusive. CRP is a non-specific and commonly used biomarker for inflammatory response. Hepatic production of CRP is increased upon stimulation by various cytokines derived from innate immune response evoked by myocardial ischemia and infarction [30]. We also observed a significant correlation between peak levels of CK-MB and CRP, implying a causative relation of the scale of infarct mass and subsequent inflammatory response. Indeed, others have also reported association of CRP levels and indexed infarct size [31, 32]. Previous clinical studies have shown that higher levels of CRP are associated with adverse prognosis in MI patients [33] including CR. Experimental studies on mice also showed the significance of infarct size in relation to loss of wall tensile strength and CR incidence [34]. Further support of inflammatory mechanism comes from our finding of higher inflammatory cell counts in CR relative to non-CR patients.

We found that the majority of CR patients (68%) arrived hospital within 24 h after symptom onset, a proportion higher than that of non-CR STEMI patients, albeit by logistic regression analysis excluded the time from symptom onset to hospital as an independent risk factor. The likely reason for the early admission of CR versus non-CR patients is due to the severity of symptom per se in patients with CR forcing them to seek medical assistance. Meanwhile, the rural location of residence of patients constitutes an independent risk factor of CR in our study. The possible explanation is that excessive time was required to arrive central hospitals to those patients who live in remote regions, and that common knowledge of MI was insufficient to those patients. Relevant to this is the limited availability of PCI especially pPCI in rural hospitals.

A significant body of knowledge has been generated by preclinical studies on the mechanism and therapeutic intervention of CR. These studies were entirely conducted in the mouse as the only laboratory species that develop CR post transmural MI like human patients [8, 22, 35]. Mechanistically, CR occurred in mice within a single onset peak timing (days 3–5) together with wall thinning and ventricular dilatation [22], simulating the human type-III rupture but without CR event within the first 24 h, which differs from our clinical finding. In the mouse model of MI, the infarct size and scale of inflammatory response are pivotal determinants of CR, observations in keeping with the findings from the present study [8, 36]. Regional inflammation results in accumulation of proteinases, particularly matrix metalloproteinase-9, responsible for the breakdown of existing collagen networks leading to reduced tensile strength of the infarcted wall [37–39]. Therapeutically, studies on mice have revealed successful inhibition of CR by anti-inflammatory therapies [40], or use of some currently routine medications like anti-platelet drugs, ARB or ACEI [35]. Collectively, our findings on patients with CR are supported in part by studies in the mouse model of CR regarding significance of infarct size, age [41], inflammation [38, 40] and histopathology of type-III CR [8, 22]. Further research is required to illustrate the mechanism of early CR and test therapeutic interventions.

Our study has a few limitations that are worth to be discussed. This was a retrospective observational study consisting data from a single center, and hence had a limited number of CR cases. Some of laboratory or angiographic data were unable to obtain in those patients who had CR at admission. Furthermore, the diagnosis of CR was based on echocardiographic images and clinical characteristics, but without pathologic (autopsy) validation.

## Conclusion

CR remains as a major cause of death in patients with MI and that the clinical features of CR patients such as risk factors, time-course, clinical presentation and mortality remain largely the same relative to the pre-PCI era. Furthermore, in mice with MI, reperfusion therapy completely prevent CR. 

## Data Availability

All data generated or analyzed during this study are included in this published article.

## Abbreviations

CR: Cardiac rupture
FWR: Free wall rupture
VSR: Ventricular septum rupture
MI: Myocardial infarction
STEMI: ST-elevation myocardial infarction
PCI: Percutaneous coronary intervention
pPCI: Primary PCI
IR: Ischemia-reperfusion
hs-CRP: High-sensitive C-reactive protein
HB: Hemoglobin
WBC: White blood cell
FDP: Fibrinogen degradation products
CK-MB: Creatine kinase-MB
ACEI: Angiotensin converting enzyme inhibitors
ARB: Angiotensin receptor blockers
IABP: Intra-aortic balloon pumping
ECMO: Extracorporeal membrane oxygenation
OR: Odds ratios
CI: Confidence intervals
