# Correction to: Cardiac rupture complicating acute myocardial infarction: the clinical features from an observational study and animal experiment

**DOI:** 10.1186/s12872-020-01713-9

**Published:** 2020-10-08

**Authors:** Qun Lu, Ping Liu, Jian-Hua Huo, Yan-Ni Wang, Ai-Qun Ma, Zu-Yi Yuan, Xiao-Jun Du, Ling Bai

**Affiliations:** 1 Department of Cardiovascular Medicine, First Affiliated Hospital, School of Medicine of Xi’an Jiaotong University, No.277 Yanta West Road, Xi’an, Shaanxi 710061 P. R. China; 2 Experimental Cardiology Lab, Baker Heart and Diabetes Institute, 75 Commercial Road, Melbourne, Victoria 3004 Australia; 3 College of Basic Medical Sciences, Xi’an Jiaotong University Health Science Center, Xi’an, Shannxi Province P. R. China

**Correction to: BMC Cardiovasc Disord (2020)**

**https://doi.org/10.1186/s12872-020-01683-y**

Following publication of the original article [1], the authors identified errors in Figs. 4 and 5. The correct figures are given below:

**Fig. 4:**
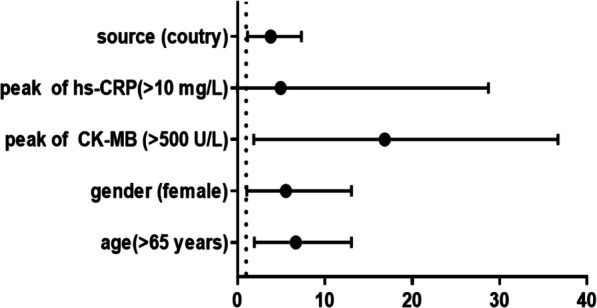
Results of the multivariate logistic regression analysis of risk factors and the incidence of CR

**Fig. 5:**
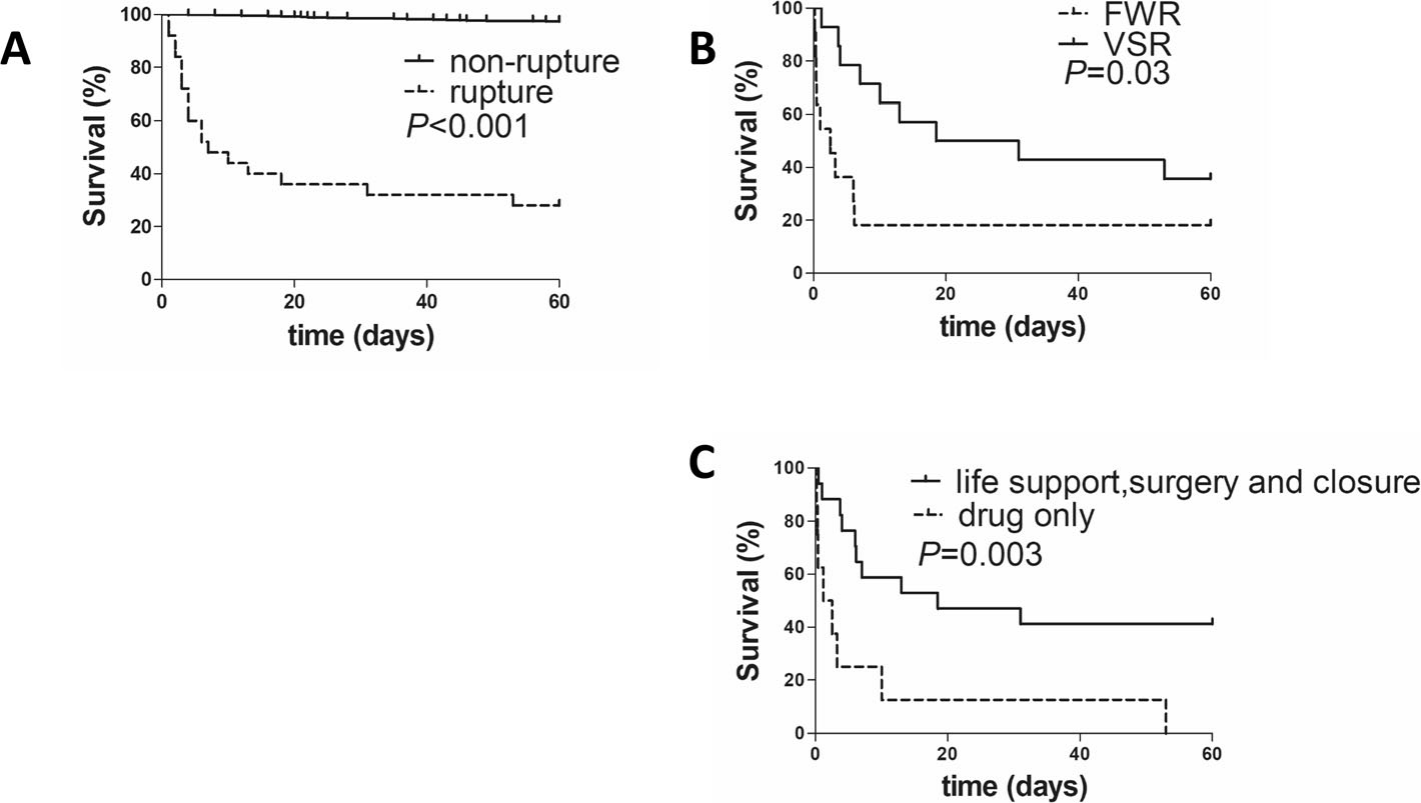
Survival curves of patients with cardiac rupture. **a** compared between in STEMI patients with and without CR. **b** compared between FWR group and VSR group. **c** compared between drug treatment only and life support, surgery and closure in CR. FWR: free wall rupture, VSR: ventricular septum rupture
